# Serum exosomal DDX17 in the diagnosis and action mechanism of hepatocellular carcinoma

**DOI:** 10.3389/fcell.2026.1897138

**Published:** 2026-10-01

**Authors:** Defa Huang, Minghong Zhao, Meijin Liu, Tao Chen, Zihao Chen, Yuhuan Xie, Haiyang Yu, Shuqian Zhong, Junyun Huang, Dingyu Rao

**Affiliations:** 1 Laboratory Medicine, First Affiliated Hospital of Gannan Medical University, Ganzhou, China; 2 Laboratory Medicine, Guizhou Aerospace Hospital, Zunyi, China; 3 Laboratory Medicine, People’s Hospital of Ganzhou Economic Development Zone, Ganzhou, China; 4 The First School of Clinical Medicine, Gannan Medical University, Ganzhou, China; 5 Department of Cardiothoracic Surgery, The First Affiliated Hospital of Gannan Medical University, Ganzhou, China

**Keywords:** biomarker, DDX17, exosomes, hepatocellular carcinoma, proteomics

## Abstract

**Background:**

This study aimed to explore DDX17 protein expression as a diagnostic biomarker of hepatocellular carcinoma (HCC) and its roles in the occurrence and development of HCC.

**Methods:**

The proteomics sequencing method was applied to study nine samples of exosomes separated from HCC tissues and corresponding normal non-cancerous tissues. We also performed nanoflow cytometry analysis of DDX17 expression in serum-derived exosomes from 102 HCC patients, 58 persons with liver cirrhosis (LC), and 80 healthy donors (HDs). The feasibility of DDX17 as a diagnostic and prognostic biomarker was evaluated using receiver operating characteristic (ROC) curves. The effects of HCC-derived exosomal DDX17 on LX-2 cell proliferation, migration, and epithelial–mesenchymal transition (EMT) were evaluated through cell proliferation, colony formation, wound healing, transwell migration, and western blotting assays.

**Results:**

We found that exosomes produced by HCC cells had much higher levels of DDX17 than those produced by the adjacent non-cancerous tissues. ROC analysis showed that exosomal DDX17 has a strong ability to distinguish HCC with both high sensitivity and high specificity. If the exosomal DDX17 result is negative, then we expect it to be a new kind of diagnostic biomarker. Moreover, we observed that DDX17-positive exosome subtypes isolated from the HepG2 cell line increased cell proliferation, migration, invasion, and EMT in the LX-2 cell line.

**Conclusion:**

DDX17-positive exosomes are potential diagnostic biomarkers of HCC and are expected to promote tumor growth, migration, invasion, and EMT.

## Introduction

Primary liver cancer is an insidious yet common disease. It is characterized by rapid progression of illness, low rates of surgical resection, and few treatment options for humans, which greatly jeopardize human health and life expectancy (Gao et al., 2020). Hepatocellular carcinoma (HCC) accounts for 80%–90% of all primary liver cancer cases, is highly aggressive, develops rapidly, and recurs easily. Although diagnostic and therapeutic strategies are available, the 5-year survival rate of HCC patients is only 12.1%, which highlights its “more proliferative and low survival” trend from statistical results (Zeng et al., 2018). Screening for patients with liver cancer includes monitoring serum alpha-fetoprotein (AFP) levels, imaging, and histological examination (Benson et al., 2017; Benson et al., 2019). Patients who are screened solely based on abnormal AFP levels do not show good sensitivity and specificity for reliable HCC prediction. Furthermore, imaging cannot detect targets smaller than 1 cm. Although tissue biopsy can provide a correct diagnosis, it is invasive and can lead to metastasis through the needle used for the procedure (Maluccio and Covey). Therefore, new biomarkers are needed to improve the accuracy of HCC diagnosis.

Exosomes are vesicles with a double membrane structure that are roughly 30–150 nm in diameter and are released from living cells (Tkach et al., 2016). They have different components, such as proteins, nucleic acids, and lipids, which represent the types of cells from which they originate (Verma et al., 2025a; Verma et al., 2023). Currently, the sources of exosomes can be divided into three categories: cellular cultures, bodily fluids, and tissues. Tissue-derived exosomes are vesicles released from most cell types found within the tissue microenvironment and provide more accurate representations of the pathophysiological traits and behaviors of tissues as they maintain their three-dimensional structure and cellular characteristics (Camino et al., 2020; Chen et al., 2020; Jang et al., 2019; Jia et al., 2024).

DEAD-box helicase 17 (DDX17) is a member of the ATP-dependent RNA helicase family that controls RNA synthesis and metabolism (Linder et al., 1989; Rocak and Linder, 2004). Notably, DDX17 is receiving attention because it is important for the operation and maintenance of other cells in cancers of the lungs, breasts, and colon (Li et al., 2017; Alqahtani et al., 2016; Shin et al., 2007). DDX17 is upregulated in many cancer patients (Xue et al., 2019; Zhou et al., 2022). There is increasing evidence that DDX17 overexpression is highly correlated with stem-cell stemness, remodeling, and malignant transformation in HCC (Feng et al., 2024). Approximately 80%–90% of HCC cases are caused by continuous inflammation and fibrosis resulting from chronic liver disease with a history of viral hepatitis or non-alcoholic steatohepatitis (NASH) (Toh et al., 2023). DDX17 promotes the occurrence of hepatitis B virus (HBV)-related HCC by improving HBV transcription and replication (Dong et al., 2022). Furthermore, other studies have shown that DDX17 plays an important role in regulating inflammatory gene expression in NASH. DDX17 has been observed to increase the production of proinflammatory cytokines, such as TNF-α, IL-6, and CCL2, thereby worsening liver steatosis and fibrosis while promoting the activation of M1 macrophages (Ning et al., 2024); this study also showed that DDX17 could be a useful marker in the diagnosis of HCC and that it is connected with disease onset and development (Verma et al., 2025b). Herein, we performed a proteomic analysis of exosomes from HCC tissues and noted enhanced DDX17 expression; this result was also confirmed in serum-derived exosomes from patients with HCC. In addition, our findings show that the exosomes from HCC promote proliferation, migration, invasion, and epithelial–mesenchymal transition (EMT) of tumor cells via DDX17.

## Materials and methods

### Data mining

The Cancer Genome Atlas (TCGA) repository (https://www.cancer.gov/tcga) provides information on whole transcriptomes and associated clinicopathological data for liver cancer. Differential expression analysis of DDX17 was conducted using the Gene Expression Profiling Interactive Analysis (GEPIA) database (http://gepia.cancer-pku.cn/index.html), along with liver cancer tissue samples (tumor group, n = 369) from TCGA and normal tissue samples (normal group, n = 50). The Tumor Immune Estimation Resource (TIMER) database (http://timer2.compbio.cn/timer1/) based on TCGA was used to analyze the Kaplan–Meier survival curves of DDX17 expression and explore how these levels are related to patient prognosis.

### Tissue and serum samples

From January to August 2024, we collected tissue and paired paracancerous tissue samples from nine HCC patients at the First Affiliated Hospital of Gannan University of Medical Sciences, along with serum samples from 102 untreated HCC patients, 58 persons with liver cirrhosis (LC), and 80 healthy donors (HDs). The inclusion criteria were as follows: patients diagnosed with primary HCC through postoperative pathological examination; age ranging from 18 to 75 years; patients who underwent radical liver resection (R0 resection) and had not received any neoadjuvant therapy or previous treatment for liver cancer before the operation; patients with Child–Pugh liver function classification grades A or B; patients with Eastern Cooperative Oncology Group physical condition scores of 0 or 1. The exclusion criteria were as follows: non-HCC cases or patients who had undergone radiotherapy, chemotherapy, or other antitumor treatments; history of other tumors or metastatic tumors in other organs; patients with incomplete clinical data (including age and sex); patients whose surgery was canceled or who did not receive a definitive pathological diagnosis postoperatively; patients with a history of blood transfusion within 30 d; patients with a history of anesthesia within 7 d. All patients underwent computed tomography examination, and their clinical signs, symptoms, and features were extracted from the electronic medical records. After examination, the HDs were ruled out as having any malignant, metabolic, or immune-related diseases. After being fully informed of the purpose and content of the study, all participants received a complete clinical diagnosis and treatment; the participants completed written informed consent forms, and the study protocols abided by the principles of the Declaration of Helsinki. This project was approved by the Ethical Committee on Scientific Research of the First Affiliated Hospital of Gannan Medical University.

### Cell culture and lentiviral infection

The HepG2 cell line was obtained from Wuhan Ponsure Life Science and Technology Co., Ltd. (third generation), MHCC-97H cells were obtained from Shanghai Yihe Applied Biotechnology Co., Ltd. (third generation), Huh7 cells were procured from Wuxi Xinrun Biotechnology Co., Ltd. (third generation), and LX-2 cells were sourced from Sichuan Biou Biotechnology Co., Ltd. (second generation). All cell lines were cultured in Dulbecco’s modified Eagle medium (DMEM; Gibco, United States) supplemented with 10% fetal bovine serum (FBS; ExcellBio, Uruguay) and 1% penicillin/streptomycin (Solarbio, China). The cells were cultured in dishes in a humidified incubator at 37 °C and 5% CO_2_. The full-length cDNA of human DDX17 was inserted into the pCDH-CMV-MCS-EF1-Puro lentiviral vector provided by Shanghai Genechem Co., Ltd. The pLKO.1 lentiviral vector was used to clone a short hairpin RNA (shRNA) targeting DDX17 with the sequence 5′-GCA​UCU​GAC​CCG​UGC​UAU​U-3' (shDDX17). Lentiviral infection was used to establish the DDX17 knockdown cell line. The HepG2 cells (5 × 10^4^) were seeded in 6-well plates, and the lentivirus was introduced for 16 h once the cells had adhered to the surface. Puromycin (2 μg/mL) screening was performed after 72 h to establish stable DDX17 knockdown in the HepG2 cells.

### Exosome isolation

Tissue exosome isolation: The tissue samples were divided into 1–2 mm pieces and placed in 2 mL of RPMI-1640 medium containing 40 U/mL of collagenase and 40 U/mL of deoxyribonuclease I as supplements. The mixture was then placed on a shaker and incubated at 37 °C for 30 min. After digestion, the supernatant was passed through a 70-μm cell strainer and centrifuged at 300*g* for 10 min. Next, the supernatant was centrifuged at 2,000*g* for an additional 20 min before being ultracentrifuged at 10,000*g* for 20 min and filtered through a 0.22-μm membrane. The supernatant was carefully removed after further ultracentrifugation of the filtrate at 110,000*g* for 42 min using a Beckman (Optima-Max-Tl, TLA-110, K-factor = 13) instrument. The obtained pellets were resuspended in 1 mL of phosphate-buffered saline (PBS) solution. The exosome suspension was further processed using a size-exclusion chromatography (SEC) column to isolate high-purity exosomes (Liaoning Rengen Biosciences Co., Ltd.). The detailed procedure is as follows. For the SEC column designed for a 1-mL sample volume, a pipette was used to first remove the PBS above the collection plate. Then, the bottom cap of the column was opened and 1.0 mL of the exosome suspension was added to the collection plate. Next, a 15-mL centrifuge tube was placed beneath the column to collect the first fraction. After the entire sample had passed through the collection plate, an appropriate volume of PBS was added (calculated as 8 mL minus the sample volume); once all the liquid had flowed through and no more liquid exited the outlet, the collection of this fraction was considered complete. The first fraction, which corresponded to a void volume of approximately 8 mL, did not contain exosomes. After continuous elution by adding PBS in 1.0 mL increments, each fraction was collected in a 1.5-mL centrifuge tube separately. The next fraction was collected after each 1.0 mL of PBS had fully passed through and no liquid remained at the outlet. Thus, each fraction collected was 1.0 mL in volume. The exosomes were primarily found in fractions 2–4, totaling a volume of approximately 3.0 mL. The final sample was collected in a 4.0-mL ultrafiltration tube with a 100-kDa cutoff (Merck Millipore, UFC810096, Germany). All centrifugation steps were carried out at 4 °C.

Serum exosome isolation: The collected blood samples were placed in coagulation-promoting tubes and centrifuged at 1,500*g* for 20 min to separate the cellular components from the debris. The supernatant was then transferred and centrifuged again at 3,000*g* for 15 min to isolate the serum. When prompt separation of exosomes was unnecessary, the extracted serum was stored at −80 °C for later processing. Next, 400 μL of plasma was moved to a 2-mL microcentrifuge tube, diluted with 1.6 mL of PBS, and centrifuged at 10,000*g* for 30 min to separate the components. The supernatant was collected and subjected to ultracentrifugation at 110,000*g* for 64 min. After carefully removing the supernatant, the pellets were resuspended in PBS and subjected to a second ultracentrifugation step at 110,000*g* for 64 min. The isolated exosomes were obtained by resuspending the final pellet in 100 μL of PBS after removing the supernatant. Finally, the exosomes were purified and stored at −80 °C for later use. All centrifugation procedures were carried out at a temperature of 4 °C.

FBS was centrifuged at 100,000*g* for 16 h at 4 °C and then filtered through a 0.22-μm-pore filter (Millipore, Germany) to obtain “exosome-depleted FBS” (Thery et al., 2006). The cells were then cultured in DMEM supplemented with 10% of the exosome-depleted FBS for 2 d until reaching 80%–90% confluence. Subsequently, 50 mL of the cell culture medium was collected, centrifuged at 1,000*g* for 10 min to eliminate cell debris, and centrifuged further at 10,000*g* for 20 min to remove the larger vesicles. The exosomes were pelleted by centrifuging the supernatant at 110,000*g* for 50 min using a Beckman centrifuge (United States). These exosomes were resuspended in PBS and subjected to another round of ultracentrifugation at 110,000*g* for 50 min to collect the final pellet. The exosome pellet was resuspended in 100 µL of PBS for further analysis. All centrifugation steps were performed at 4 °C.

### Exosome identification by transmission electron microscopy

The collected exosomes were combined, transferred onto Formvar carbon-coated 200-mesh copper grids, and allowed to adsorb at room temperature for 10 min. After adsorption, the exosomes were negatively stained with 3% phosphotungstic acid at room temperature for 5 min. The grids were air-dried after staining and analyzed using a Hitachi H-7600 transmission electron microscope operated at an acceleration voltage of 80.0 kV.

### Western blotting

The proteins were resolved using 12% SDS-polyacrylamide gel electrophoresis and subsequently transferred onto a polyvinylidene fluoride membrane with a microporous structure. The membrane was then blocked using 5% skim milk powder for 1 h and incubated overnight at 4 °C with antibodies against Alix (catalog no. ab186728, Abcam, United States), CD63 (catalog no. ab134045, Abcam), TSG101 (catalog no. ab133586, Abcam), GM130 (catalog no. ab52649, Abcam), DDX17 (catalog no. ab205719, Abcam), N-cadherin (catalog no. ab321897, Abcam), E-cadherin (catalog no. ab227639, Abcam), Vimentin (catalog no. ab217673, Abcam), and GAPDH (catalog no. 60004-1-Ig, Proteintech, China). The secondary antibody was a horseradish-peroxidase-conjugated goat anti-rabbit/mouse IgG antibody (catalog no. A00001-2/SA00001-1, Proteintech, China).

### Nanoparticle flow cytometry analysis

The exosome samples were analyzed for particle concentration, size distribution, and surface proteins using a nanoflow cytometer (nFCM) from Xiamen, China. A standard curve was established on the nFCM detector using silica nanoparticle size standards to determine the particle size. The exosome samples were measured for size distribution using 20 μL of the solution. FITC-labeled mouse anti-human DDX17 antibody (catalog no. CSB-PA846106LC01HU, CUSABIO, China) was used to detect the surface proteins. In brief, samples containing a mixture of 50 μL of exosomes and 5 μL of the anti-DDX17 antibody were incubated for 30 min at 37 °C. Following incubation, the exosomes were centrifuged at 110,000*g* for 30 min at 4 °C and washed twice. After the final wash, the exosome pellet was resuspended in 50 μL of PBS, and the nFCM system was used to determine the percentage of DDX17-positive exosomes. We set the threshold as the mean value of the fluorescence signal from the blank control plus three times the standard deviation, thereby eliminating the background and spontaneous fluorescence effectively. Based on the maximum sum of sensitivity and specificity of the patient sample (at the maximum Youden index), we determined the threshold for detecting the DDX17-positive exosomes as 12.4%.

### PKH67-labeled exosomes

The isolated exosomes were labeled with a PKH67 green fluorescence labeling kit (Sigma) according to manufacturer instructions. The LX-2 cells were seeded into 6-well plates; after the cells had adhered completely, a membrane dye (prepared using the medium in the ratio of 1:1,000) was added for staining at 37 °C for 15 min. Then, 100 μL of the serum exosomal vesicles and 4 μL of PKH67 were diluted in 1 mL of Dilution C and mixed evenly; this mixture was incubated for 4 min at room temperature. Next, 2 mL of 1% bovine serum albumin (BSA) was added to bind the PKH67. The resulting samples were centrifuged twice at 110,000*g* for 70 min to remove the excess PKH67. The PKH67-labeled exosomes were resuspended in DMEM and added to the LX-2 cells. After co-incubation for 12 h, the cells were fixed with 2% paraformaldehyde for 10 min and blocked with a mounting medium (with DAPI). Lastly, images were captured using a confocal fluorescence microscope (Zeiss).

### Trypsin treatment and mass spectrometry analysis

The exosomal protein levels were measured using a Pierce BCA Protein Quantification Kit (Thermo Fisher Scientific, Inc.); each protein sample was mixed with 3 μL of trypsin at 1 μg/μL and 500 μL of 100 mM triethyl ammonium bicarbonate buffer before being digested overnight at 37 °C. After digestion, each sample was mixed with an equal volume of 1% formic acid and centrifuged at 12,000*g* for 5 min at room temperature. The supernatant was carefully applied to a C18 desalting column in a stepwise manner, followed by three washes with 1 mL of a washing solution containing 0.1% formic acid and 4% acetonitrile; subsequently, the sample was eluted twice using 0.4 mL of an elution buffer composed of 0.1% formic acid and 75% acetonitrile. The eluents were then mixed and freeze-dried. The lyophilized protein powder was reconstituted in 10 μL of 0.1% formic acid in water (solvent A) and subsequently loaded onto a custom-made C18 Nano-Trap column (2 cm × 75 μm, 3 μm). A homemade analytical column (15 cm × 150 μm, 1.9 μm) was used to separate the peptides with a mobile phase consisting of 0.1% formic acid in 80% acetonitrile (solvent B). The sample was eluted at a flow rate of 600 nL/min, and the concentration of solvent B was increased from 6% to 100% over 60 min. A Q Exactive HF-X mass spectrometer (Thermo Fisher Scientific, Inc.) with a Nanospray Flex™ (ESI) ion source was used to analyze the peptides by applying a spray voltage of 2.3 kV. The raw mass spectrometry data were searched against the UniProt human proteome database using MaxQuant software with an integrated Andromeda search engine. Protein quantification was performed using label-free quantification (LFQ) intensities generated using MaxQuant; for each protein, the LFQ intensity was log-transformed, and proteins with fewer than three valid values in at least one group were excluded from subsequent analyses. The fold change (FC) for each protein was calculated as the ratio of the average log_2_-transformed LFQ intensity between the exosome and control groups. Proteins with |log_2_FC| > 0.585 (corresponding to FC > 1.5) and adjusted *p*-value < 0.05 were considered to be significantly differentially expressed.

### Immunohistochemistry

Tissue section fixed in formalin and embedded in paraffin were prepared for immunohistochemical analysis using a streptavidin-peroxidase kit (Proteintech, China) and monoclonal antibodies against DDX17 (catalog no. ab180190, Abcam). Positive cells were quantified using a scoring system in the range of 0–4, with 0 indicating no positivity and 4 representing 76%–100% positive staining. The positively stained areas were assessed on a scale of 0–3, with the scores reflecting weak (1), moderate (2), and strong (3) relative staining intensities. The total score was obtained by multiplying the intensity and percentage scores. A fully automated digital pathology slide scanner (KFBIO, China) was used to capture the corresponding images.

### Analysis of tumor markers

The AFP tumor marker levels were analyzed using an Elecsys Immunoassay Analyzer (COBAS-E-602; Roche, Switzerland). The cutoff value for the AFP level was set at 10 ng/mL.

### Cell proliferation assay

The LX-2 cells (5 × 10^3^ per well with five replicates for each group) was plated in 96-well culture plates and exposed to exosomes (100 μg/mL) derived from various groups or treated with PBS. A group lacking cells was used as the blank control. After 24 h, 10 μL of the cell counting kit-8 (CCK-8) reagent (Biosharp, China) was added to each well containing 100 μL of the culture medium. A microplate reader was used to measure the absorbance of each well at 450 nm after 1.5 h of incubation at 37 °C.

### Colony formation assay

The LX-2 cells (8 × 10^2^ per well with three replicates for each group) were plated in 96-well culture plates and exposed to exosomes (100 μg/mL) from various groups or PBS. The cells were placed in an incubator at 37 °C and cultured for 2 weeks under constant temperature conditions. The culture was stopped and the initial medium was removed and discarded before washing the cells three times with PBS. The cells in each well were fixed in 4% paraformaldehyde for 30 min. The cells were then stained with a crystal violet solution for 8 min. The cell colonies were photographed and counted during the final stage.

### Scratch wound healing assay

The LX-2 cells (5 × 10^5^ per well with three replicates per group) were plated in 6-well plates and cultured at 37 °C until attachment was observed. A sterile 200-μL pipette tip was used to create linear scratch wounds on the cell monolayer. After washing with PBS to eliminate any detached cells, the wells were treated with exosomes (100 μg/mL) from various groups or an equivalent volume of PBS. Images of the LX-2 cells were captured at 0 and 24 h post-wounding. The migration rate (%) was calculated as [(wound area at 0 h − wound area at 24 h)/wound area at 0 h] × 100%.

### Transwell assay

In the transwell assay, the cells were suspended in serum-free medium (5 × 10^4^ cells per well using three replicates for each group) and added to the upper chamber of the 24-well transwell (Corning, United States) with 8-μm pore filters. The cells were then treated with exosomes (100 μg/mL) derived from various groups or PBS. The lower chamber of each transwell was filled with 650 μL of complete medium supplemented with 15% FBS. After 24 h, the cells adhering to the upper side of the filter membrane were cleared, whereas those that migrated to the lower side were briefly stained with 0.1% crystal violet for a few minutes. An optical microscope was used to capture the images of the cells.

### Statistical analysis

GraphPad Prism 8.0 software was used for the statistical analyses, and all data are shown as mean ± standard error (SE). The t-test was used for comparisons between two groups, while one-way ANOVA was used to compare more than one group. MedCalc18.0 was used to create the receiver operating characteristic (ROC) curve, and statistical significance was established as *p* < 0.05.

## Results

### Characterization of tissue-derived exosomes

The aim of this study is to not only identify good diagnostic markers for HCC but also determine the steps in the progression of HCC. Figure 1A shows the complete workflow of this study. The exosomes display a bilayer structure and concave hemispherical shape when viewed under a transmission electron microscope (Figure 1B). The nanoflow cytometry analysis showed that the isolated exosomes were 50–200 nm in diameter (Figure 1C). Western blotting showed the presence of exosomal markers, such as CD63, Alix, and TSG101, but not the negative marker GM130 in the isolated exosomes (Figure 1D). These results match the criteria established by the International Extracellular Vesicle Society for defining exosomes.

**FIGURE 1:**
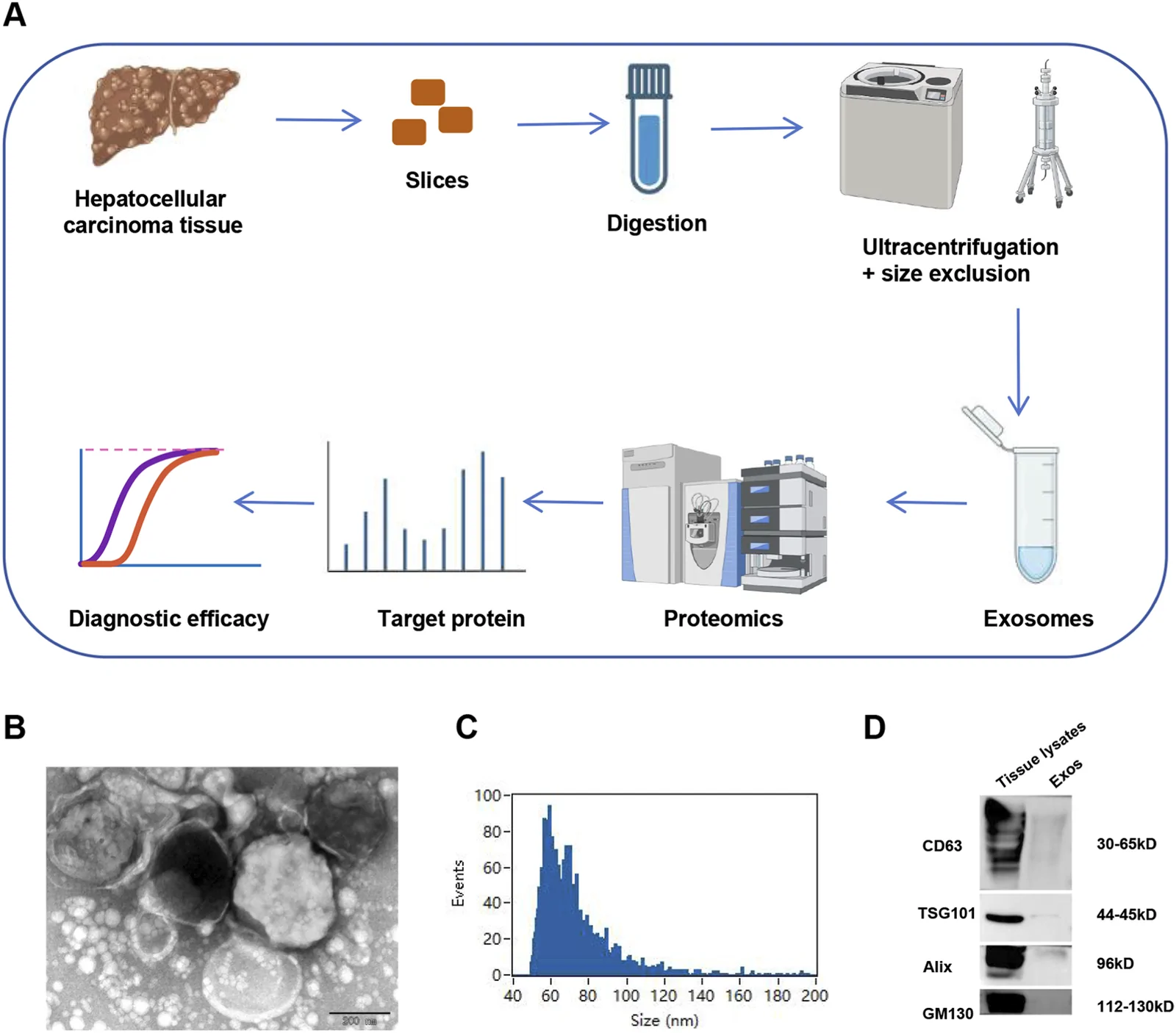
Characterization of the isolated exosomes. **(A)** Schematic workflow of the study. **(B)** Representative transmission electron microscopy (TEM) image of exosomes showing round or cup-like shapes; scale bar: 200 nm. **(C)** Quantitative size distribution profile of the isolated exosomes in suspension as determined by nanoflow cytometry; the peak diameter is approximately 80–100 nm, and the majority of particles fall within the canonical exosome size range of 50–200 nm. **(D)** Western blotting results of CD63, TSG101, Alix, and GM130 exosomal biomarkers.

### Proteomic analysis of exosomes derived from HCC and adjacent tissues of patients

Proteomic sequencing analysis was performed on the exosomes isolated from the tumors and adjacent normal tissues of nine patients with HCC after purification. In the tumor-regulated protein program, a total of 235 differentially expressed proteins were identified with FC > 1.5; among these proteins, 60 were upregulated (26%) and 175 were downregulated (74%). Hierarchical clustering analysis was then performed on the differentially expressed proteins using the log_2_FPKM method; the resulting heat map had columns representing different samples and rows representing different proteins, and all data were normalized. High expressions are shown in red, whereas low expressions are shown in green (Figures 2A,B). As shown in Figure 2C, DDX17 protein expression was significantly upregulated in the exosomes obtained from HCC tissues. In addition, we performed differential analysis using the GEPIA database to compare the expression levels of the DDX17 gene between liver cancer tissue samples (tumor group, n = 369) and normal liver tissue samples (normal group, n = 50) from TCGA database. As shown in the figure, DDX17 was more highly expressed at the transcriptome level in tumor tissues than the adjacent normal liver tissues (Figure 2D).

**FIGURE 2:**
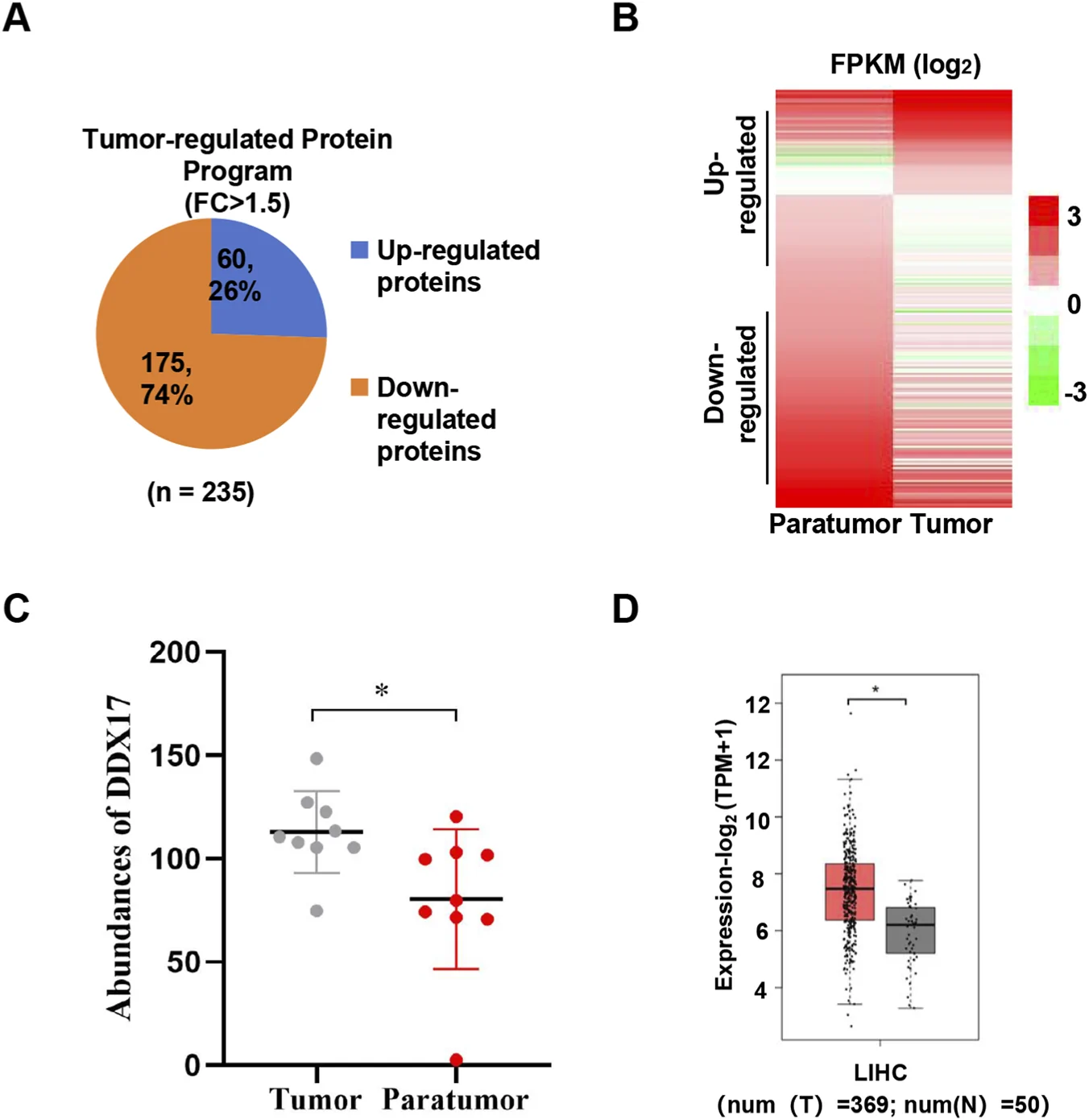
Proteomics analysis to identify differentially targeted molecules. **(A)** Pie chart showing the statistical results of the proteomics sequencing analysis. **(B)** Clustering heatmap results from the proteomics sequencing analysis. **(C)** DDX17 expression levels in exosomes obtained from HCC tissues (T) and neighboring non-cancerous tissues (P). **(D)** Gene expression analysis results of DDX17 in liver cancer patients and normal individuals based on information from the GEPIA database. *p < 0.05.

### Expression of DDX17 and survival prognosis in HCC

We observed the expressions of DDX17 in HCC samples and correlated it with the tumor stage in nine cases for paired HCC and adjacent non-tumor tissue samples via immunohistochemistry. Tumor staging was performed using the BCLC tumor staging system. From the statistical data, we observed that DDX17 expression increased with tumor development compared to that in the surrounding tissues (Figures 3A,B). Kaplan–Meier survival analysis was used to study the association between DDX17 expression and patient prognosis. Analysis of clinical data based on the TIMER database revealed that among the 371 patients with liver cancer, 69 were in the low DDX17 expression group while 302 were in the high DDX17 expression group. The Kaplan–Meier analysis revealed no statistically significant association between DDX17 expression level and overall survival (log-rank *p* = 0.08) or disease-free survival (log-rank *p* = 0.7), although a non-significant trend was observed toward poorer survival in the high DDX17 expression group for overall survival (Figures 3C,D). We then used the nFCM to detect DDX17 in the serum-derived exosomes from each group. We also analyzed the correlation between the level of DDX17-positive exosomes and clinical characteristics of patients with HCC and found that these were related to the tumor stage and AFP level. However, no significant association was observed between the level of DDX17-positive exosomes and patient characteristics (age, gender, etc.) (Table 1). Serum exosome analysis showed that the DDX17-positive exosome levels were significantly higher in the HCC group than in the HD and LC groups. The combination of DDX17 and AFP levels was a more accurate predictor than using each marker individually, as shown in Figure 4B. In patients who were negative for AFP, we observed that the DDX17 level could still be used as a good diagnostic characteristic (Figure 4C; Table 2).

**FIGURE 3:**
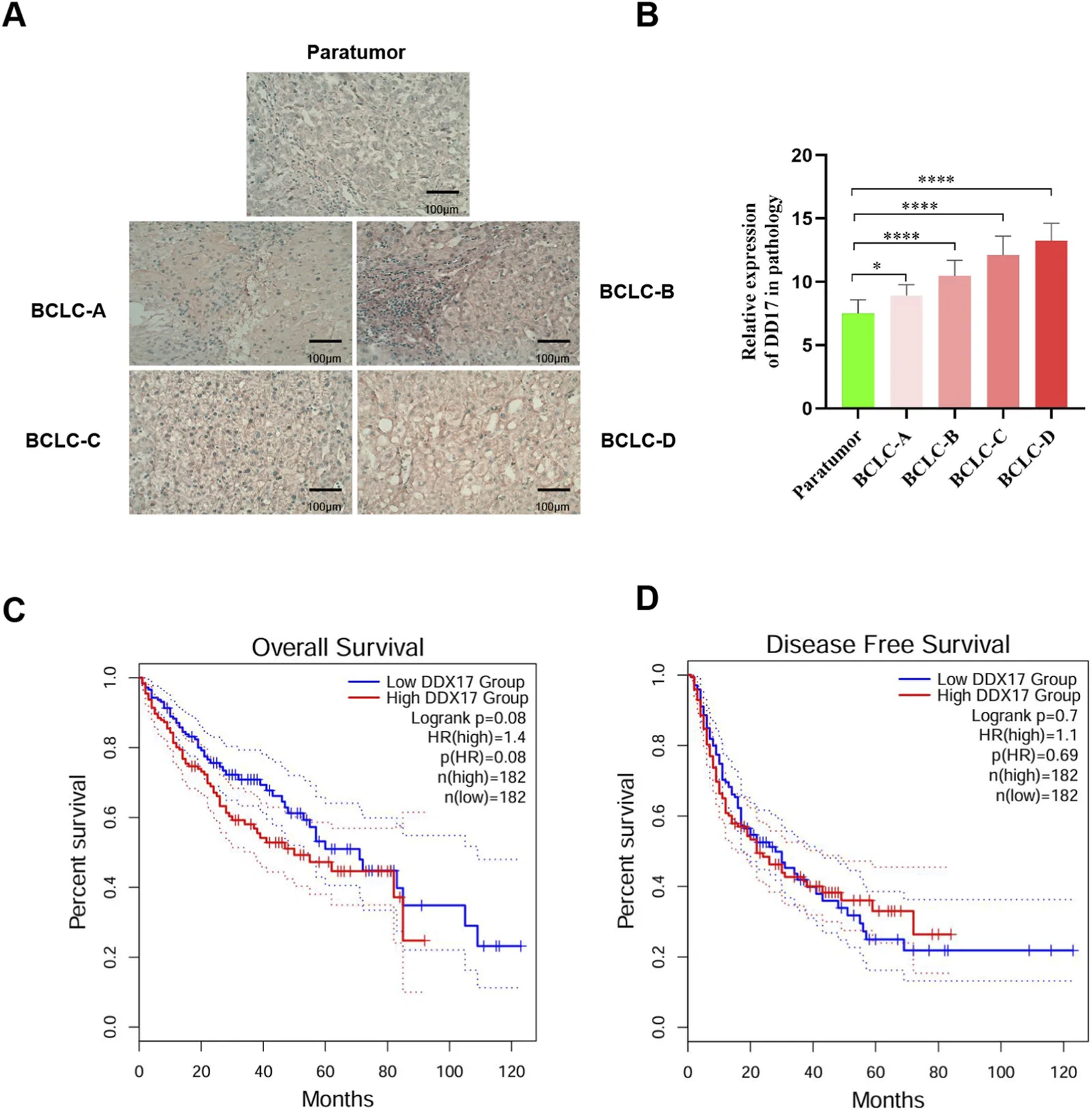
Investigation of DDX17 expression levels in liver cancer tissues and their association with patient survival outcomes. **(A)** Representative images of DDX17 expression at different levels in HCC and paired non-tumor tissues; scale bar: 100 μm. **(B)** Relative expressions of DDX17 protein in patients with different stages of HCC. Kaplan–Meier curves for **(C)** overall survival (OS) and **(D)** disease-free survival (DFS) of HCC patients from TCGA database stratified by high (red) and low (blue) expression levels of the DDX17 mRNA; the optimal cutoff for DDX17 expression was determined using maximally selected rank statistics. The hazard ratio (HR) and log-rank *p*-value are shown on each graph. *p < 0.05; ****p < 0.0001.

**TABLE 1:** Serum DDX17-positive exosome levels and clinical characteristics of patients with hepatocellular carcinoma (HCC).

Characteristic		Number of cases	DDX17-positive exosomes (%)	*p*-value
Age (years)	<50	48	15.22 ± 2.21	0.730
	≥50	54	14.68 ± 3.15	
Gender	Male	50	16.20 ± 3.22	0.690
	Female	52	15.26 ± 2.98	
Hepatitis B viral infection	Absent	24	14.45 ± 2.93	0.342
	Present	78	15.72 ± 3.25	
Alpha-fetoprotein (AFP; ng/mL)	≤200	39	14.78 ± 4.12	0.034
	>200	63	19.26 ± 3.97	
BCLC status	A–B	46	13.95 ± 2.68	0.041
	C–D	56	18.22 ± 3.36	

**FIGURE 4:**
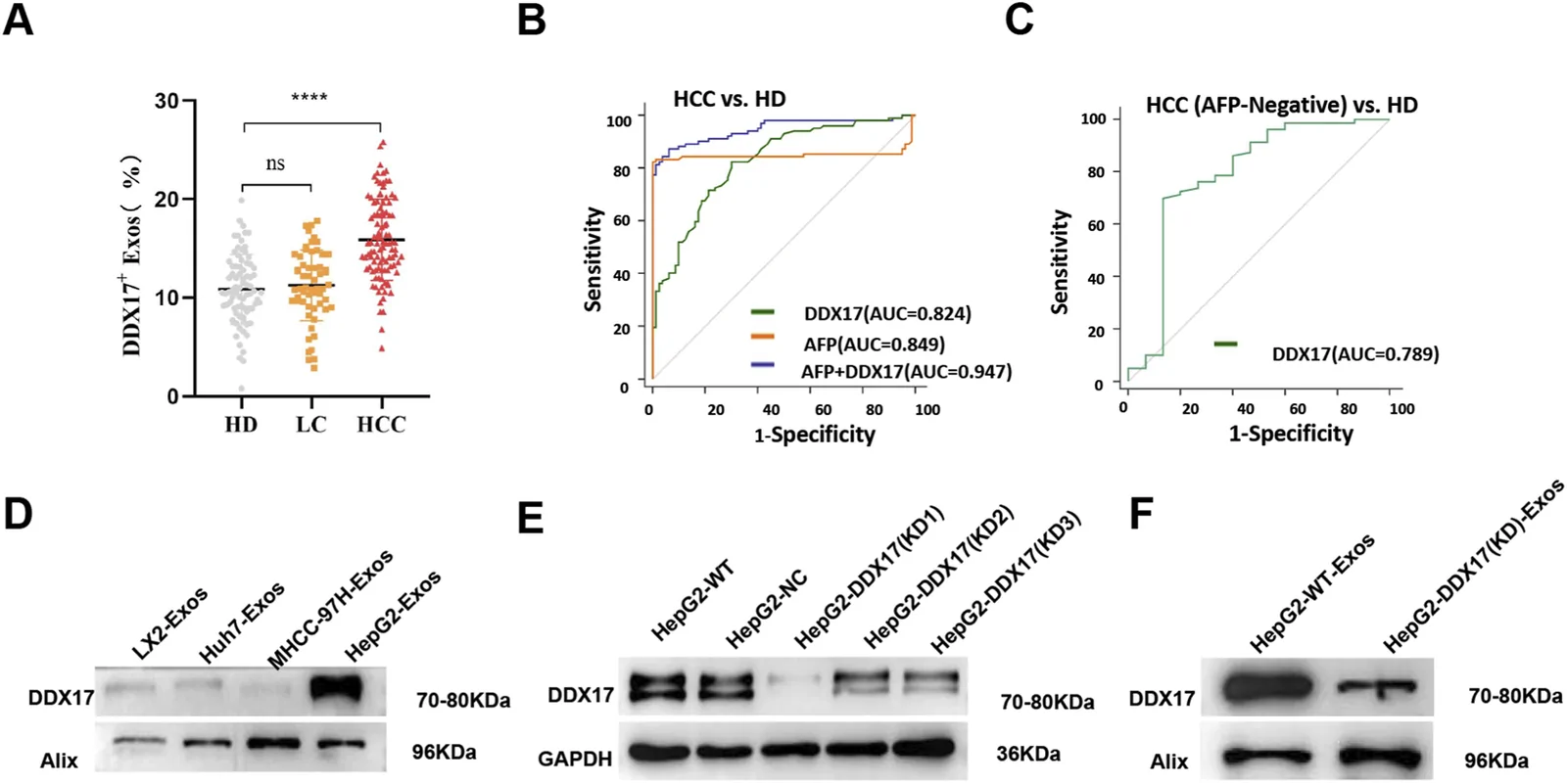
Expression and diagnostic performance evaluations of DDX17-positive exosomes in serum. **(A)** The ratios of DDX17-positive to total exosomes (DDX17^+^/total) were significantly different between the healthy donor (HD) and liver cirrhosis (LC)/HCC groups. **(B)** Diagnostic potential of DDX17-positive exosomes in distinguishing HCC patients from HDs. **(C)** Diagnostic potential of DDX17-positive exosomes in distinguishing AFP-negative HCC patients from HDs. **(D)** DDX17 expression levels in exosomes derived from the supernatant of human hepatic stellate cells (LX2-Exos) and three hepatoma cell lines (Huh7-Exos, MHCC-97H-Exos, and HepG2-Exos). **(E)** Protein expression levels of DDX17 in HepG2 parental cells, HepG2 cells transfected with a negative control lentivirus (HepG2-WT), and HepG2 cells transfected with a DDX17 knockdown lentivirus (HepG2-DDX17(KD)). **(F)** DDX17 expression levels in exosomes derived from HepG2-WT and HepG2-DDX17(KD) cells. ****p < 0.0001.

**TABLE 2:** Diagnostic efficacies of models in differentiating hepatocellular carcinoma (HCC) cases from healthy donors (HDs).

Variable	Area under the curve	95% confidence interval	Sensitivity (%)	Specificity (%)	Youden index	*p*-value
HCC vs. HD						
DDX17	0.824	0.761–0.876	82.35	70.00	0.52	<0.0001
AFP	0.849	0.789–0.898	82.35	100	0.82	<0.0001
DDX17 + AFP	0.947	0.904–0.975	87.25	93.75	0.81	<0.0001
HCC (AFP-negative) vs. HD						
DDX17	0.789	0.693–0.866	70.00	86.67	0.56	0.0004

## Exosomes derived from HCC promote tumor proliferation, migration, invasion, and EMT through DDX17

We compared DDX17 expression levels in exosomes from normal stellate cells (LX2-Exos) with those from three hepatoma cell lines (Huh7-Exos, MHCC-97H-Exos, and HepG2-Exos) to explore the exosomal expression characteristics in different hepatoma cell lines. The findings showed that DDX17 expression was significantly higher in HepG2-Exos than LX2-Exos (Figure 4D). Considering these findings, we chose the HepG2 cell line that exhibited the highest DDX17 expression level for subsequent gene knockdown experiments (Figure 4E). The knockdown of DDX17 expression in HepG2 cells led to a decrease in DDX17 level in the exosomes isolated from these cells (HepG2-DDX17(KD)-Exos) (Figure 4F). Next, we examined exosome uptake by the LX-2 cells; following labeling with the PKH67 fluorescent dye, the exosomes were incubated with LX-2 cells. The labeled HepG2-Exos were efficiently taken up by LX-2 after exposure for 12 h (Figure 5A). We isolated exosomes from both normal HepG2 cells (HepG2-WT-Exos) and those with DDX17 knockdown (HepG2-DDX17(KD)-Exos) to investigate the possible biological roles of DDX17-positive exosomes derived from HepG2 cells. Exosomes from the normal HepG2 cell line promoted proliferation, migration, and invasion of LX-2 cells; however, these functions decreased when exosomes isolated from DDX17 knockdown cells were used (Figures 5B–H). Additionally, this shows that DDX17 is related to exosomal function. Western blotting also showed that LX-2 cells treated with HepG2-DDX17(KD)-Exos for 48 h had significantly less E-cadherin with more N-cadherin and Vimentin (Figures 5I,J). These results indicate that exosomes from HCC affect the proliferation, migration, and EMT of LX-2 cells in a DDX17-dependent manner and emphasize the important role of exosome-mediated communication in changing the tumor microenvironment.

**FIGURE 5:**
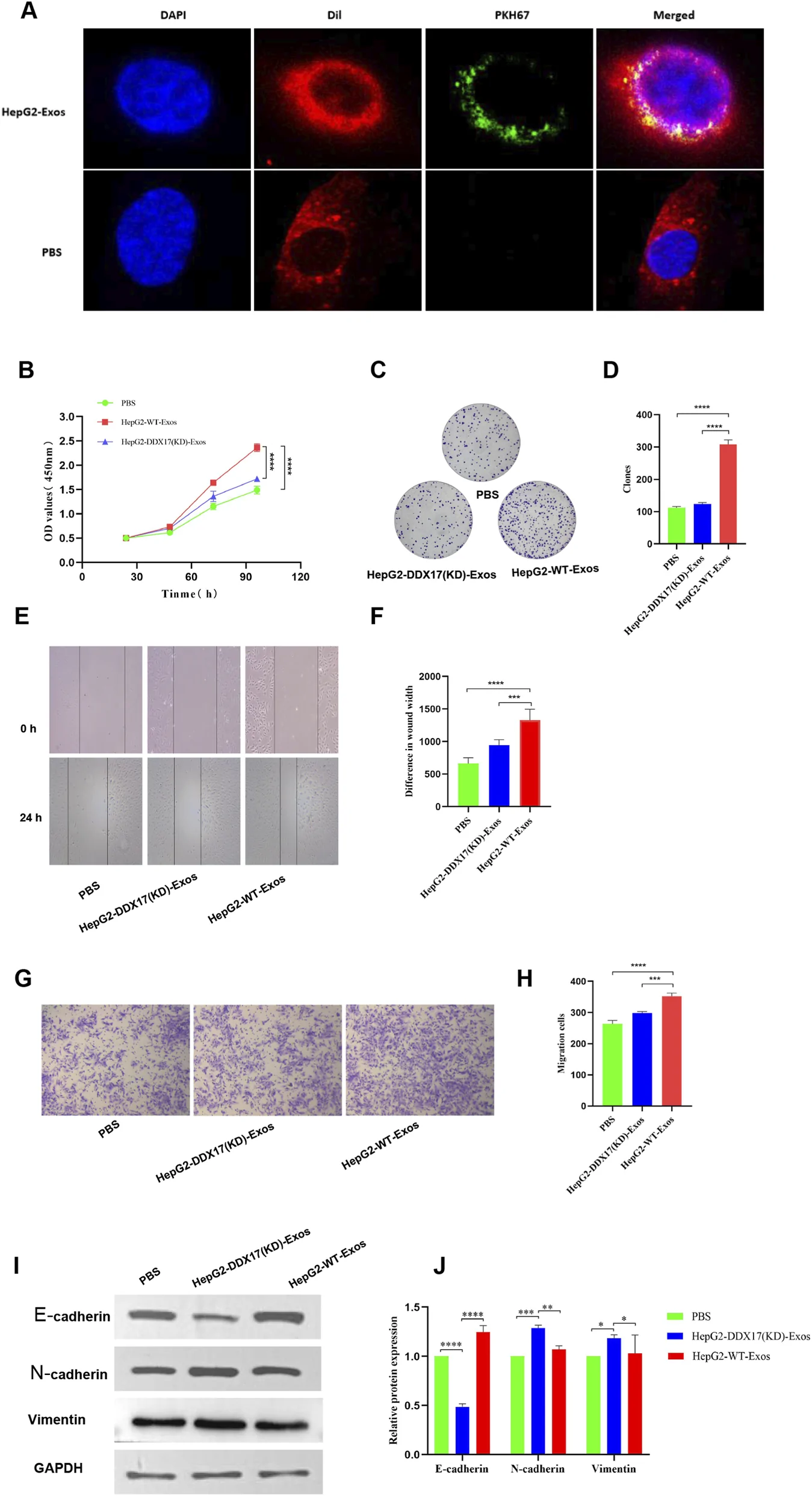
Exosomes derived from HCC promote tumor proliferation, migration, invasion, and epithelial–mesenchymal transition through DDX17. **(A)** Uptake of PKH67-labeled HepG2-Exos by LX-2 cells as analyzed by immunofluorescent microscopy; blue: DAPI; red: Dil; green: PKH67. **(B)** Proliferation analysis results of LX-2 cells treated with phosphate-buffered saline (PBS), HepG2-WT-Exos, and HepG2-DDX17(KD)-Exos (treatment time: 24, 48, 72, and 96 h). **(C, D)** Cloning analysis results of LX-2 cells treated with PBS, HepG2-WT-Exos, and HepG2-DDX17(KD)-Exos (treatment time: 2 weeks); original image magnification: 10×. **(E, F)** Wound healing analysis results of LX-2 cells treated with PBS, HepG2-WT-Exos, and HepG2-DDX17(KD)-Exos (treatment time: 24 h); original image magnification: 40×. **(G, H)** Invasion analysis results of LX-2 cells treated with PBS, HepG2-WT-Exos, and HepG2-DDX17(KD)-Exos (treatment time: 24 h); original image magnification: 40×. LX-2 cells were treated with exosomes for 24 h, followed by **(I)** western blotting analysis using antibodies and **(J)** quantification of protein levels as shown. The data are derived from at least three independent experiments. *p < 0.05; **p < 0.01; ***p < 0.001; ****p < 0.0001.

## Discussion

Liver cancer is a disease in which the affected persons do not show or experience any early signs until the disease has mostly progressed (Llovet et al., 2021). The disease itself is difficult to treat and has unfortunate outcomes. However, the most serious threat to global public health is its very high mortality rate, which is related to poor tumor progression, high predilection for metastasis, and frequent recurrence. HCC has many causes, and differences in the genetic makeup of the affected persons make it difficult to determine the condition early on (Fu and Wang, 2018). Therefore, HCC patients tend to be diagnosed late and have a rather grim outlook; the overall 5-year survival rate is approximately 10.1% (Zeng et al., 2015). Thus, early discovery of HCC is better for the affected persons. The older methods of diagnosis tend to focus on the overall conditions of the affected persons using imaging for targeted detection of the problem areas. With the development of high-throughput sequencing technologies and availability of increasing amounts of multi-omics data, a large number of diagnostic models based on gene expression have been developed. A diagnostic model based on two genes linked to the tumor mutational load has been developed, which produced area under the curve (AUC) values of 0.928 and 0.950 for the training and validation groups, respectively; the authors also created a model using two DNA-methylation-driven genes with AUC values of 0.978 and 0.981 (Long et al., 2019). Moreover, Cheng et al. (2022) developed a machine learning diagnostic model that displayed high performance with AUCs of 0.987 and 0.992 for different cohorts.

In our study, we used different ultracentrifugation methods to separate the exosomes from HCC tissues and their corresponding adjacent non-tumor tissues from the nine cases. Using proteomic sequencing technology and integrated bioinformatics database analysis, we found that DDX17 is highly expressed in HCC. Regarding clinical prognosis, survival analysis did not demonstrate a statistically significant prognostic value of exosomal DDX17 in this cohort. This trend warrants further investigation in larger and well-powered cohorts. We used nanoflow cytometry to test the serum-derived exosomal DDX17 in HDs and patients with HCC and LC. Thus, we can conclude that DDX17 expression levels differ greatly in patients with HCC. In the ROC curve analysis, combining DDX17-positive exosome and AFP levels produced more accurate diagnoses than using their individual levels. The DDX17-positive exosomes have a notable diagnostic ability for HCC in AFP-negative patients, with an AUC value of 0.789.

We performed western blotting to assess DDX17 expression levels in different liver cancer cell lines and determine the role of DDX17-positive exosomes in HCC. Accordingly, we found that DDX17 expressions were very high in three different hepatoma cell lines (Huh7, MHCC-97H, and HepG2) and in the exosomes derived from these cells compared to normal liver cells (LX-2). To study how DDX17-positive exosomes affect LX-2 cell proliferation, migration, and invasion and to understand how hepatoma-cell-derived exosomal DDX17 changes the behavior of LX-2 cells, we performed the CCK-8 proliferation and colony formation assays. The proliferation of LX-2 cells was enhanced by the exosomes of HepG2-WT cells compared to the PBS control group. In the wound healing and transwell migration experiments, the migration and invasion of LX-2 cells were significantly higher than those in the control group, indicating that these cells had greater migration and invasion abilities after treatment with HepG2-WT exosomes. These exosomes enhanced LX-2 cell proliferation, migration, and invasion via DDX17. To determine whether DDX17-positive exosomes affect EMT, western blotting was performed on the HepG2-WT exosomes and PBS control. The HepG2-WT exosome treatment reduced E-cadherin and Vimentin expressions while increasing N-cadherin expression. Together, these findings verify that exosomes control EMT via DDX17. Previous studies have shown that DDX17 promotes carcinogenesis in HCC. DDX17 forms a functional complex with β-catenin and NF-κB, facilitating their nuclear transport and thereby enhancing transcriptional activation of the proinflammatory chemokine CXCL8 (Liu et al., 2025). This implies that tumor-cell-derived DDX17 modulates the tumor immune microenvironment by recruiting immunosuppressive populations, including tumor-associated neutrophils and myeloid-derived suppressor cells, via the CXCL8 signaling axis. Notably, recent studies have delineated a tightly regulated reciprocal relationship between DDX17 and EMT: the transcription factor TCF4 directly binds to the DDX17 promoter to induce its expression; conversely, through its RNA helicase domain, DDX17 promotes nuclear accumulation of β-catenin. This nuclear β-catenin associates with TCF4 to form a transcriptionally active complex that further amplifies DDX17 transcription, establishing a self-reinforcing β-catenin/TCF4/DDX17 positive feedback loop (Lv et al., 2025). Building on this mechanistic insight, we hypothesized that exosomal DDX17 delivered to LX-2 hepatic stellate cells or HCC cells may cooperatively accelerate EMTs and fibrotic remodeling within the tumor microenvironment.

Although this study offers novel insights, certain limitations warrant further consideration: the use of a single-cell model may not fully capture the heterogeneity of HCC; we considered only a relatively superficial mechanistic investigation of exosomal DDX17, which requires further in-depth molecular dissection; the lack of *in vivo* experimental verifications limit the translational relevance of our findings; the absence of an independent diagnostic validation cohort restricts the generalizability of our conclusions regarding the diagnostic potential of exosomal DDX17. Further research is necessary to clarify the specific action mechanisms of exosomal DDX17, including extension to multiple cell lines and patient-derived samples to verify its functional roles through *in vivo* xenografting and *in situ* animal models. Multimodal analysis could also be used to explore other action mechanisms of exosomal DDX17 in the occurrence and development of liver cancer (Gao et al., 2025). We suggest conducting mechanistic studies involving RNA immunoprecipitation and luciferase reporter gene assays to reveal the downstream signaling pathways, along with establishing an independent multicenter cohort to evaluate the diagnostic and prognostic value of exosomal DDX17 in clinical settings.

## Conclusion

Our results indicate that serum-derived DDX17-positive exosomes may serve as diagnostic biomarkers for detecting HCC, particularly in AFP-negative patients. Furthermore, DDX17-positive exosomes originating from HCC cells may contribute to tumor growth, migration, invasion, and EMT.

## Data Availability

The proteomics data generated in this study are associated with ongoing unpublished research and are currently being further analyzed. Requests for access to these datasets should be directed to the corresponding author.

## References

[B1] Alqahtani H. Gopal K. Gupta N. Jung K. Alshareef A. Ye X. (2016). DDX17 (P72), a Sox2 binding partner, promotes stem-like features conferred by Sox2 in a small cell population in estrogen receptor-positive breast cancer. Cell. Signal. 28 (2), 42–50. 10.1016/j.cellsig.2015.11.004 26569340

[B2] Benson A. B. D'Angelica M. I. Abbott D. E. Abrams T. A. Alberts S. R. Saenz D. A. (2017). NCCN guidelines insights: hepatobiliary cancers, version 1.2017. J. Natl. Compr. Canc Netw. 15 (5), 563–573. 10.6004/jnccn.2017.0059 28476736 PMC5557008

[B3] Benson A. B. D'Angelica M. I. Abbott D. E. Abrams T. A. Alberts S. R. Anaya D. A. (2019). Guidelines insights: hepatobiliary cancers, version 2.2019. J. Natl. Compr. Canc Netw. 17 (4), 302–310. 10.6004/jnccn.2019.0019 30959462

[B4] Camino T. Lago-Baameiro N. Martis-Sueiro A. Couto I. Santos F. Baltar J. (2020). Deciphering adipose tissue extracellular vesicles protein cargo and its role in obesity. Int. J. Mol. Sci. 21 (24), 9366. 10.3390/ijms21249366 33316953 PMC7764772

[B5] Chen X. D. Zhao J. Yan Z. Zhou B. W. Huang W. F. Liu W. F. (2020). Isolation of extracellular vesicles from intestinal tissue in a mouse model of intestinal ischemia/reperfusion injury. Biotechniques 68 (5), 257–262. 10.2144/btn-2019-0159 32090587

[B6] Cheng B. Zhou P. Chen Y. (2022). Machine-learning algorithms based on personalized pathways for a novel predictive model for the diagnosis of hepatocellular carcinoma. BMC Bioinforma. 23 (1), 248. 10.1186/s12859-022-04805-9 35739471 PMC9219178

[B7] Dong M. L. Wen X. He X. Ren J. H. Yu H. B. Qin Y. P. (2022). HBx mediated increase of DDX17 contributes to HBV-related hepatocellular carcinoma tumorigenesis. Front. Immunol. 13, 871558. 10.3389/fimmu.2022.871558 35784274 PMC9243429

[B8] Feng F. Wu J. Chi Q. Wang S. Liu W. Yang L. (2024). Lactylome analysis unveils Lactylation-Dependent mechanisms of stemness remodeling in the liver cancer stem cells. Adv. Sci. (Weinh) 11 (38), e2405975. 10.1002/advs.202405975 39099416 PMC11481176

[B9] Fu J. Wang H. (2018). Precision diagnosis and treatment of liver cancer in China. Cancer Lett. 412, 283–288. 10.1016/j.canlet.2017.10.008 29050983

[B10] Gao Y. X. Yang T. W. Yin J. M. Yang P. X. Kou B. X. Chai M. Y. (2020). Progress and prospects of biomarkers in primary liver cancer. Int. J. Oncol. 57 (1), 54–66. 10.3892/ijo.2020.5035 32236573

[B11] Gao Z. Yan X. Wang-Sattler R. Covic M. Yu G. Ge F. (2025). “Metabolomics reveals reasons for the efficacy of acupuncture in migraine patients: the role of anaerobic glycolysis and mitochondrial citrate in migraine relief,”, 5. Cham, Switzerland, 707–728. 10.1007/s43657-024-00205-6 Phenomics 6 41675281 PMC12886611

[B12] Jang S. C. Crescitelli R. Cvjetkovic A. Belgrano V. Olofsson Bagge R. Sundfeldt K. (2019). Mitochondrial protein enriched extracellular vesicles discovered in human melanoma tissues can be detected in patient plasma. J. Extracell. Vesicles 8 (1), 1635420. 10.1080/20013078.2019.1635420 31497264 PMC6719261

[B13] Jia W. T. Xiang S. Zhang J. B. Yuan J. Y. Wang Y. Q. Liang S. F. (2024). Jiedu recipe, a compound Chinese herbal medicine, suppresses hepatocellular carcinoma metastasis by inhibiting the release of tumor-derived exosomes in a hypoxic microenvironment. J. Integrative Medicine 22 (6), 696–708. 10.1016/j.joim.2024.10.002 39521704

[B14] Li K. Mo C. Gong D. Chen Y. Huang Z. Li Y. (2017). DDX17 nucleocytoplasmic shuttling promotes acquired gefitinib resistance in non-small cell lung cancer cells via activation of beta-catenin. Cancer Lett. 400, 194–202. 10.1016/j.canlet.2017.02.029 28259822

[B15] Linder P. Lasko P. F. Ashburner M. Leroy P. Nielsen P. J. Nishi K. (1989). Birth of the D-E-A-D box. Nature 337 (6203), 121–122. 10.1038/337121a0 2563148

[B16] Liu H. Gao X. Zhang W. Fu X. Zhang J. Yuan Q. (2025). DDX17-Mediated upregulation of CXCL8 promotes hepatocellular carcinoma progression via Co-activating β-catenin/NF-κB complex. Int. J. Biol. Sci. 21 (3), 1342–1360. 10.7150/ijbs.104165 39897048 PMC11781183

[B17] Llovet J. M. Kelley R. K. Villanueva A. Singal A. G. Pikarsky E. Roayaie S. (2021). Hepatocellular carcinoma. Nat. Rev. Dis. Prim. 7 (1), 6. 10.1038/s41572-020-00240-3 33479224 PMC13537433

[B18] Long J. Chen P. Lin J. Bai Y. Yang X. Bian J. (2019). DNA methylation-driven genes for constructing diagnostic, prognostic, and recurrence models for hepatocellular carcinoma. Theranostics 9 (24), 7251–7267. 10.7150/thno.31155 31695766 PMC6831284

[B19] Lv C. Luo N. Luo Y. Zhang Q. Cao X. Cheng J. (2025). β‐Catenin/TCF4 is required for DDX17‐Induced epithelial–mesenchymal transition and metastasis in hepatocellular carcinoma. MedComm – Oncol. 4 (3), e70039. 10.1002/mog2.70039

[B20] Maluccio M. Covey A. (2012). Recent progress in understanding, diagnosing, and treating hepatocellular carcinoma. CA Cancer J. Clin. 62 (6), 394–399. 10.3322/caac.21161 23070690

[B21] Ning D. Jin J. Fang Y. Du P. Yuan C. Chen J. (2024). DEAD-box helicase 17 exacerbates non-alcoholic steatohepatitis via transcriptional repression of cyp2c29, inducing hepatic lipid metabolism disorder and eliciting the activation of M1 macrophages. Clin. Transl. Med. 14 (2), e1529. 10.1002/ctm2.1529 38303609 PMC10835191

[B22] Rocak S. Linder P. (2004). DEAD-box proteins: the driving forces behind RNA metabolism. Nat. Rev. Mol. Cell. Biol. 5 (3), 232–241. 10.1038/nrm1335 14991003

[B23] Shin S. Rossow K. L. Grande J. P. Janknecht R. (2007). Involvement of RNA helicases p68 and p72 in colon cancer. Cancer Res. 67 (16), 7572–7578. 10.1158/0008-5472.CAN-06-4652 17699760

[B24] Thery C. Amigorena S. Raposo G. Clayton A. (2006). Isolation and characterization of exosomes from cell culture supernatants and biological fluids. Curr. Protoc. Cell Biol. 10.1002/0471143030.cb0322s30 18228490

[B25] Tkach M. Thery C. (2016). Communication by extracellular vesicles: where we are and where we need to Go. Cell 164 (6), 1226–1232. 10.1016/j.cell.2016.01.043 26967288

[B26] Toh M. R. Wong E. Y. T. Wong S. H. Ng A. W. T. Loo L. H. Chow P. K. (2023). Global epidemiology and genetics of hepatocellular carcinoma. Gastroenterology 164 (5), 766–782. 10.1053/j.gastro.2023.01.033 36738977

[B27] Verma N. Khare D. Poe A. J. Amador C. Ghiam S. Fealy A. (2023). MicroRNA and protein cargos of Human limbal epithelial cell-derived exosomes and their regulatory roles in limbal stromal cells of diabetic and non-diabetic corneas. Cells 12 (21), 2524. 10.3390/cells12212524 37947602 PMC10649916

[B28] Verma N. Arora S. Singh A. K. Ahmed J. (2025a). Unlocking the potential of exosomes ‘extracellular vesicles’: drug delivery advancements and therapeutics in ocular diseases. RSC Pharm. 2 (6), 1201–1226. 10.1039/d5pm00097a

[B29] Verma N. Arora S. Singh A. K. Kumar A. (2025b). Extracellular vesicle-associated miRNAs in cornea health and disease: diagnostic potential and therapeutic implications. Targets 3 (4), 32. 10.3390/targets3040032

[B30] Xue Y. Jia X. Li C. Zhang K. Li L. Wu J. (2019). DDX17 promotes hepatocellular carcinoma progression via inhibiting Klf4 transcriptional activity. Cell Death Dis. 10 (11), 814. 10.1038/s41419-019-2044-9 31653828 PMC6814716

[B31] Zeng H. Zheng R. Guo Y. Zhang S. Zou X. Wang N. (2015). Cancer survival in China, 2003-2005: a population-based study. Int. J. Cancer 136 (8), 1921–1930. 10.1002/ijc.29227 25242378

[B32] Zeng H. Chen W. Zheng R. Zhang S. Ji J. S. Zou X. (2018). Changing cancer survival in China during 2003-15: a pooled analysis of 17 population-based cancer registries. Lancet Glob. Health 6 (5), e555–e567. 10.1016/S2214-109X(18)30127-X 29653628

[B33] Zhou H. Z. Li F. Cheng S. T. Xu Y. Deng H. J. Gu D. Y. (2022). DDX17-regulated alternative splicing that produced an oncogenic isoform of PXN-AS1 to promote HCC metastasis. Hepatology 75 (4), 847–865. 10.1002/hep.32195 34626132 PMC9304246

